# Perceived rhythmic regularity is greater for song than speech: examining acoustic correlates of rhythmic regularity in speech and song

**DOI:** 10.3389/fpsyg.2023.1167003

**Published:** 2023-05-26

**Authors:** Chu Yi Yu, Anne Cabildo, Jessica A. Grahn, Christina M. Vanden Bosch der Nederlanden

**Affiliations:** ^1^The Brain and Mind Institute, Western University, London, ON, Canada; ^2^Department of Psychology, Western University, London, ON, Canada; ^3^Department of Psychology, University of Toronto, Mississauga, ON, Canada

**Keywords:** rhythmic regularity, beat, speech, song, music information retrieval, periodicity, rhythm

## Abstract

Rhythm is a key feature of music and language, but the way rhythm unfolds within each domain differs. Music induces perception of a beat, a regular repeating pulse spaced by roughly equal durations, whereas speech does not have the same isochronous framework. Although rhythmic regularity is a defining feature of music and language, it is difficult to derive acoustic indices of the differences in rhythmic regularity between domains. The current study examined whether participants could provide subjective ratings of rhythmic regularity for acoustically matched (syllable-, tempo-, and contour-matched) and acoustically unmatched (varying in tempo, syllable number, semantics, and contour) exemplars of speech and song. We used subjective ratings to index the presence or absence of an underlying beat and correlated ratings with stimulus features to identify acoustic metrics of regularity. Experiment 1 highlighted that ratings based on the term “rhythmic regularity” did not result in consistent definitions of regularity across participants, with opposite ratings for participants who adopted a beat-based definition (song greater than speech), a normal-prosody definition (speech greater than song), or an unclear definition (no difference). Experiment 2 defined rhythmic regularity as how easy it would be to tap or clap to the utterances. Participants rated song as easier to clap or tap to than speech for both acoustically matched and unmatched datasets. Subjective regularity ratings from Experiment 2 illustrated that stimuli with longer syllable durations and with less spectral flux were rated as more rhythmically regular across domains. Our findings demonstrate that rhythmic regularity distinguishes speech from song and several key acoustic features can be used to predict listeners’ perception of rhythmic regularity within and across domains as well.

## Introduction

Rhythm is crucial for the perception and production of vocal communication in both music and language. In language, syllable rhythms aid in the segmentation of speech (Cutler and Butterfield, 1992; Dilley and McAuley, 2008), convey the meaning of the speaker through prosodic stress (e.g., sarcasm, Cheang and Pell, 2008), illustrate the presence of a foreign speakers’ accent (Polyanskaya et al., 2017), and support simultaneous acquisition of multiple languages in infancy (Werker and Byers-Heinlein, 2008). In music, rhythm contributes to melodic identity (Jones et al., 1987; Hébert and Peretz, 1997), enables beat perception (Povel and Essens, 1985; Parncutt, 1994), impacts perceived groove in music (Matthews et al., 2019), and provides the structure that allows synchronization with music or other people (Fitch, 2016). Rhythm is clearly an important feature for both language and music, but the way that rhythm is realized in each domain—that is, how rhythm unfolds in time—is different.

Rhythm, in both music and language, can be defined as the pattern of ‘events’ in time (McAuley, 2010; Ravignani and Madison, 2017). Events in language typically occur at the syllable level, and events in music occur at the note level. Music and language differ in how the time intervals between events are structured. In musical rhythms, events are usually structured around a beat, or an underlying pulse (Drake, 1998; McAuley, 2010). Even though individual events are not equally spaced, the intervals between events relate to the beat, which means that durations are most commonly related by small integer ratios like 1:2 (e.g., quarter note:half note). The beat in music leads to the perception that the intervals between beats are roughly the same duration (i.e., isochronous; Ravignani and Madison, 2017; Ravignani and Norton, 2017) and gives listeners the sense of periodicity, or the perception of a pattern repeating regularly at a fixed period or interval in time (Patel, 2003; Patel et al., 2005; Kotz et al., 2018). Periodicity is present in music even despite natural tempo fluctuations or expressive timing that make a strictly isochronous beat improbable in human produced music (Fraisse, 1982; Epstein, 1985; Bharucha and Pryhor, 1986). In contrast, speech rhythms do not have a beat. It is this presence of a beat that we call rhythmic regularity.

Despite a long history of searching for strictly periodic intervals at the syllable or stress level in speech, no one has found regularly repeating patterns of equal duration in speech (Grabe and Low, 2002; Patel, 2003; Patel et al., 2005; Cummins, 2012; Goswami and Leong, 2013; Brown et al., 2017). Although speech sounds are generally considered rhythmic, those rhythms are constrained to the length of the word, linguistic stress pattern, syntactic rules, or prosodic emphasis in a sentence (Cutler and Foss, 1977; Hay and Diehl, 2007; Turk and Shattuck-Hufnagel, 2013), which does not lend well to rhythmic regularity. These temporal regularities are crucial for speech intelligibility (Shannon et al., 1995) and more crucial than spectral characteristics of speech (Albouy et al., 2020). Speakers learn the typical rhythmic patterns of their language and this knowledge gives rise to temporal predictability in speech (Rosen, 1992; Hawkins, 2014; Jadoul et al., 2016; Rathcke et al., 2021), rather than any rhythmic regularities in the speech signal (Beier and Ferreira, 2018). The differences in regularity between music and language are especially salient when comparing sensorimotor synchronization to speech and song, where speech has much greater variability in the alignment of taps to syllable events in speech (30%) compared to note events song (4%, Lidji et al., 2011; Cummins, 2012; Dalla Bella et al., 2013).

In each domain, there is considerable research characterizing the degree or type of rhythmic information in the signal. These studies ask, for instance, whether language is rhythmic at all (e.g., Nolan and Jeon, 2014) or what acoustic factors contribute to the strength of perceived regularity in music (e.g., Bouwer et al., 2018). A range metrics have been used to characterize rhythm and/or regularity within each domain and, in a few cases, across domains. These metrics include the calculation of inter-onset-intervals between successive notes or syllables (e.g., stressed and unstressed IOIs; Vanden Bosch der Nederlanden et al., 2022a,b), durational contrastiveness between pairs of successive notes or syllables (Pairwise Variability Index; Grabe and Low, 2002; Patel and Daniele, 2003; Hannon, 2009; Hannon et al., 2016), the proportion of vocalic intervals in an utterance (vowel reduction; Grabe and Low, 2002; Wiget et al., 2010; Arvaniti, 2012), acoustic feature extraction using music information retrieval techniques (e.g., Lartillot and Toiviainen, 2007; Lartillot et al., 2008; Alluri and Toiviainen, 2010; Burger et al., 2013, 2014), autocorrelations to detect self-similarity in the envelope of a signal (Leong, 2012; Suppanen et al., 2019), clock timing evidence and counter-evidence (Povel and Essens, 1985), and integer multiple relatedness (Roeske et al., 2020; De Gregorio et al., 2021). These metrics have been useful within their own contexts of identifying, for example, whether a composer’s language background influenced the musical rhythms they employed (Patel and Daniele, 2003; Van Handel, 2006) or determining the strength of a beat in one musical rhythm compared to another (Henry et al., 2017; Matthews et al., 2019). However, not all speech-rhythm metrics have proven to be reliable or strong predictors of perceived speech rhythms (White and Mattys, 2007; Arvaniti, 2012; Jadoul et al., 2016). In music, the task of beat extraction is difficult (McKinney et al., 2007; Grosche et al., 2010), even if humans do it spontaneously (Grahn and Brett, 2007; Honing, 2012). The goal of the current paper is to examine whether some of the above metrics used to characterize rhythmic regularity in music or language separately can characterize the differences in rhythmic regularity *between* language and music.

Past work has examined where in the acoustic signal the beat is located in speech and song, finding consistent tapping in speech and song at p-centers (but see conflicting takes on p-centers Morton et al., 1976; *cf.* Marcus, 1981; Vos and Rasch, 1981; Pompino-Marschall, 1989; Scott, 1998; Villing et al., 2007), vowel onsets (Rathcke et al., 2021), or at peaks in the acoustic envelope (Kochanski and Orphanidou, 2008; Lartillot and Grandjean, 2019). Still others have used cochlear models of acoustic salience to find the beat location in vocally-produced songs (Ellis, 2007; Coath et al., 2009). While these approaches are germane to the current question, our goal is to determine whether acoustic features of speech and song can eventually provide evidence of rhythmic regularity—in the form of an equally-spaced, repeating pulse—in a range of communicative and non-communicative domains. For instance, there is increasing evidence that regularity is a salient feature in the sensory landscape (Aman et al., 2021), with listeners detecting regularity within a single cycle of it emerging from a random background (Southwell and Chait, 2018) or preferentially attending to a visual stream with statistical regularities despite having no conscious perception of that regularity (Zhao et al., 2013). Stimuli in studies like these are created with careful control over what features should give rise to regularity, but a wide range of natural stimuli, including non-human animal vocalizations (Kotz et al., 2018; Roeske et al., 2020; De Gregorio et al., 2021) and environmental sounds (e.g., Gygi et al., 2004; Rothenberg, 2013) also give rise to regularity in a variety of different acoustic characteristics. Our goal is to find a metric that indexes the differences in regularity between speech and song with the future goal of using this metric to detect the degree of regularity in a range of naturally occurring sounds.

Acoustic features that differentiate temporal regularity in speech and song will also feed into perceptual and cognitive questions related to how humans differentiate speech and song in development (Vanden Bosch der Nederlanden et al., 2022a,b). Rhythmic regularity is an important feature for speech-to-song or environmental sound-to-song transformations (Simchy-Gross and Margulis, 2018; Tierney et al., 2018; Rowland et al., 2019), but spectral features seem to be better predictors of a listeners’ perception of an utterance as speech or song (Hilton et al., 2022; Vanden Bosch der Nederlanden et al., 2022a,b; Albouy et al., 2023; Ozaki et al., 2023). Given the importance of rhythmic differences between and among languages for helping children acquire language (Ramus et al., 1999; Nazzi et al., 2000; Jusczyk, 2002), and for bringing about a transformation from speech to song, a clear acoustic metric of rhythmic regularity may prove useful for understanding the development of distinct domains of communication.

We address the goals in the current study by first obtaining subjective ratings of the differences in rhythmic regularity between spoken and sung utterances. After establishing this subjective metric, acoustic features of spoken and sung utterances were related to subjective ratings of rhythmic regularity to examine which features are most predictive of perceived rhythmic regularity.

## Experiment 1

### Participants

Thirty-three 18- to 24-year-old participants (16 males) participated in the study. An additional 7 people participated in the study but were excluded because they did not complete the study (*N* = 5 did not provide a rating for at least 90% of the rating trials, *N* = 2 did not pass attention checks within the survey; see Procedure). A third of participants reported taking music lessons and a third of participants self-reported being bilingual, but most participants were English monolinguals who learned English from birth (see Supplementary Table S1). About half of participants identified as white. Participants were recruited from the University of Western Ontario undergraduate psychology participant pool and were required to speak English fluently and have no known hearing deficits. All participants were compensated with course credit and provided informed consent to participate. All materials were approved by Western University’s Research Ethics Board (REB).

### Stimuli

One set of sung and spoken utterances was used for Experiment 1. We used a stimulus set generated for a different study (see Vanden Bosch der Nederlanden et al., 2022a,b). For purposes related to the previous studies’ need for acoustic control, the spoken and sung utterances were acoustically matched on several features, including the sentence texts (see Appendix A), speaker identity, total duration (utterance length), tempo (syllable rate), pitch contour, RMS amplitude, and number of syllables. In total, this stimulus set included 96 stimuli (48 unique texts), 48 spoken, 48 sung, with 3 male speakers (American and British English accents). The stimuli ranged from 1.62 to 3.86 s in length with an average of approximately 2.46 s. For details on stimulus creation please see Vanden Bosch der Nederlanden et al. (2022a).

### Procedure

Participants accessed the online study using Qualtrics (2021) and completed a regularity rating task and a background demographics questionnaire. In the rating task, participants heard each spoken or sung sentence presented in random order in a single block. The presentation order of spoken and sung utterances was not constrained, so participants could hear multiple spoken or sung utterances in a row. On each trial, participants rated each audio clip according to how rhythmically regular it sounded (see Appendix B1), using a rating scale of 1 (not very regular) to 9 (very regular). Two catch trials were randomly presented to ensure participants were paying attention. The audio in these catch trials gave explicit instructions for ratings. For example, if the catch trial audio said “This is a test trial. Please select number 3 on the slider below,” the participant should have moved the slider to 3 before proceeding to the next trial. Immediately after the rating task, participants were asked to write out their own definition of rhythmic regularity in an open text box. Participants completed a demographic background questionnaire at the end. On average, participants completed the study in 33.61 min.

### Results

**Figure 1:**
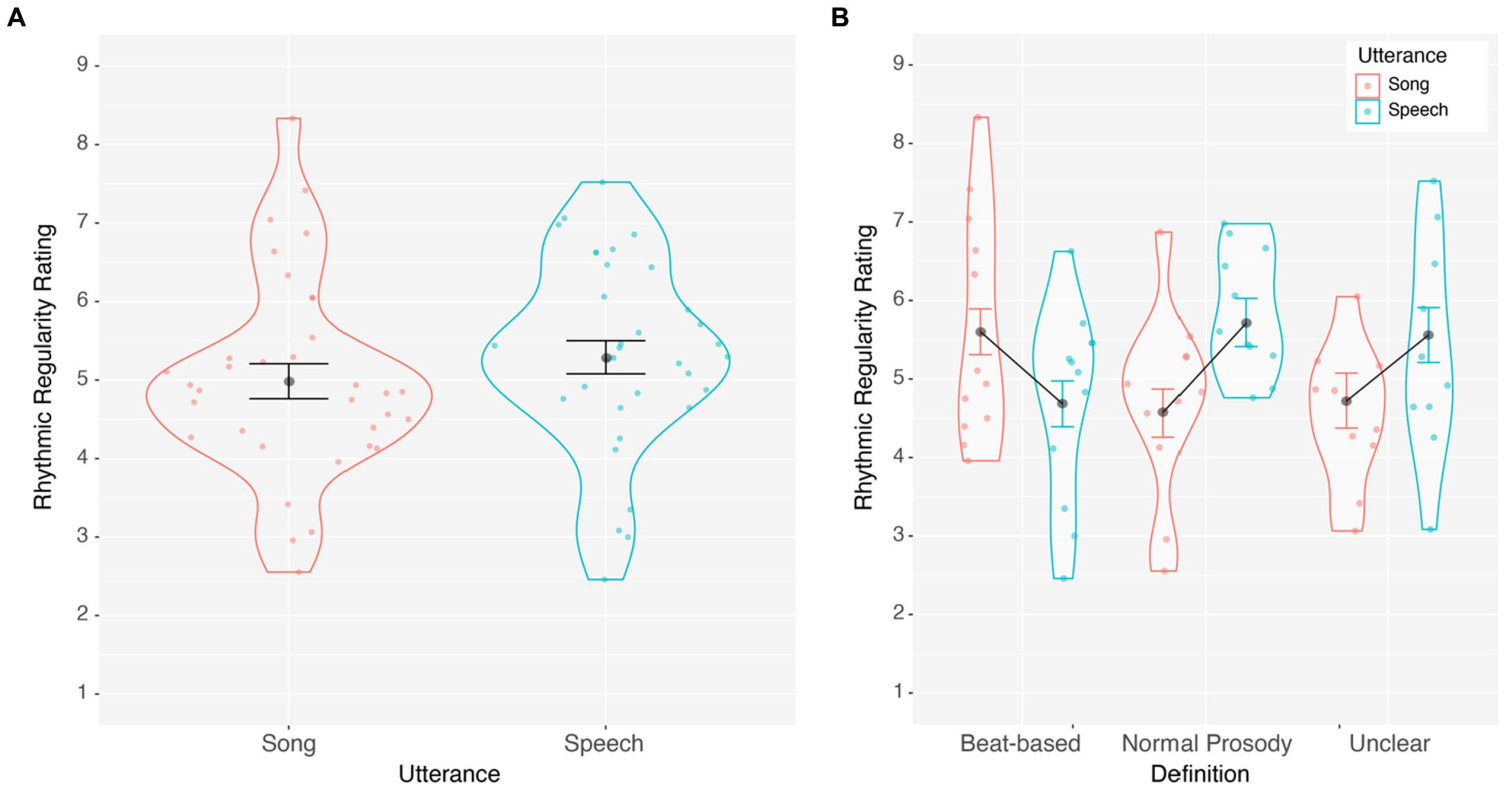
**(A)** Average rhythmic regularity rating of song and speech illustrating no difference in regularity ratings and **(B)** a significant interaction illustrating that speech and song regularity ratings were dependent on participants’ definition of rhythmic regularity. Standard Error is within-subjects error (Morey, 2008).

Rhythmic regularity ratings were averaged separately for spoken and sung utterances. Ratings were normally distributed, with skewness and kurtosis ratings between +/−3. Average ratings were submitted to a one-way repeated-measures Analysis of Variance (ANOVA) with Utterance (Speech, Song) as the main factor. As illustrated in Figure 1A, regularity ratings did not differ between speech and song, *F*(1, 32) = 1.044, *p* = 0.314, η^2^ = 0.032. However, we provided no training or guidance on what rhythmic regularity was. To capture whether participants’ definition of rhythmic regularity influenced their ratings, we thematically coded each listener’s self-reported definition of “rhythmic regularity” and identified 3 groups: beat-based, normal-prosody, and unclear definitions. Participants were grouped into beat-based definitions if they mentioned the words “beat” or “meter” and/or discussed the importance of rhythmic consistency (e.g., even spacing). Participants were grouped into normal-prosody definitions if they discussed linguistic stress, prosodic pitch, rhyme, and that regularity depended on sounding normal for conversation (e.g., normal speed/tempo/flow for speech). Finally, participants were placed in the unclear definition group if their definition was not based on acoustic factors (e.g., annoyance, familiarity), was not a definition (e.g., about what the goal of the study was), or had a definition that could be either beat or prosody based (see Supplementary Table S2). In the end, 12 listeners had beat-based definitions, 11 listeners had normal-prosody definitions, and 10 listeners had unclear definitions of rhythmic regularity. A follow-up 2 (Utterance: speech, song) by 3 (Definition: beat, prosody, unclear) ANOVA again showed no main effect of utterance type (speech vs. song), *F*(1,30) = 1.934, *p* = 0.175, η_p_^2^ = 0.061, but there was a significant interaction with definition, *F*(2, 30) = 6.606, *p* = 0.004, η_p_^2^ = 0.306. As illustrated in Figure 1B, the normal-prosody group rated speech as more rhythmically regular than song, *F*(1, 10) = 7.085, *p* = 0.024, η^2^ = 0.415, while the beat-based group rated song as more rhythmically regular than speech, *F*(1, 11) = 4.963, *p* = 0.048, η^2^ = 0.311, and the unclear group did not reliably differentiate regularity in speech and song, *F*(1, 9) = 2.846, *p* = 0.126, η^2^ = 0.240. These results suggest that the perceived rhythmic regularity of speech and song differed based on participants’, sometimes inaccurate, definition of rhythmic regularity.

## Interim discussion

Experiment 1 illustrated that participants had varying definitions of rhythmic regularity when we left it undefined and did not provide training examples. Initially it appeared that our acoustically matched stimuli did not differ in perceived rhythmic regularity, but after taking participants’ definitions into account (whether their definition was beat-based, normal-prosody, or unclear), regularity was greater for song than speech for beat-based definitions and greater for speech than song for normal-prosody definitions. Note that the normal-prosody definition group did not describe prosodic rhythmic regularity or a beat in speech, but rather participants in this group largely based their definitions only on the regular part of the term rhythmic regularity. Instead, these participants focused on how normal the speech sounded for everyday conversations. Although definition groupings explained a significant amount of variability in regularity ratings, it is also possible that the acoustic constraints placed on the stimuli reduced the differences in rhythmic regularity between spoken and sung exemplars. In this case, different profiles of regularity for speech and song in Experiment 1 may mean stimuli did not differ or only weakly differed in rhythmic regularity. We designed Experiment 2 to determine whether providing a clear definition of rhythmic regularity would shift participants’ ratings to align with the beat-based definition of rhythmic regularity we set out to examine in addition to determining whether regularity ratings were consistent across different stimulus sets.

We improved on Experiment 1 in three ways: (1) We provided a concrete rhythmic regularity rating scale “How easy would it be to tap or clap along to that clip?” (2) We provided training examples before participants began the rating task consisting of spoken and sung clips that would be easy and not easy to tap or clap to using familiar stimuli, and (3) We added a second unmatched stimulus set of spoken and sung stimuli that were not acoustically matched to examine regularity differences between unconstrained spoken and sung exemplars.

A second goal of Experiment 2 was to relate participants’ regularity ratings to acoustic features of spoken and sung exemplars. To achieve this goal, speech- and music-based acoustic features were extracted from all stimuli using Praat, MIR Toolbox, and custom music-inspired scripts (see OSF). We used standard acoustic features that are known to differ between speech and song (Vanden Bosch der Nederlanden et al., 2022a,b), as well as several features described in the introduction related to temporal regularity (see Appendix D for full feature list).

## Experiment 2

### Participants

Fifty-one participants (13 males) between the ages of 17–24 years of age participated. An additional 6 individuals participated but were excluded because they did not pass all attention checks (see Procedure). Note that one included participant passed attention checks but did not respond to 2 trials in the acoustically matched stimulus set. About a quarter of the participants reported musical training (see Supplementary Table S3). Almost a third of participants self-reported being bilingual, but most participants were English monolinguals and learned English from birth (see Supplementary Table S3). About half of participants identified as white (see Supplementary Table S3). Participants were recruited from the University of Western Ontario undergraduate psychology participant pool and were required to be English speakers and have no known hearing deficits. All participants were compensated with course credit and provided informed consent to participate. All materials were approved by Western University’s Research Ethics Board (REB).

### Stimuli

Experiment 2 included the acoustically matched stimulus set from Experiment 1 and an unmatched stimulus set created for this study. This additional stimulus set addressed the possibility that matched spoken and sung utterances did not differ on rhythmic regularity because of the constraints placed on tempo, duration, contour in their recording process. The unmatched stimulus set consisted of short clips pulled from several free sources on the internet including audiobooks.org (*N* = 15), looperman.com (*N* = 7), ccmixter.org (*N* = 12), Soundcloud.com (*N* = 2), the SiSEC database (*N* = 8; Liutkus et al., 2017), and a previous paper examining music and language comparisons (*N* = 1; Albouy et al., 2020). Podcast recordings (*N* = 15) were sampled from spotify.com under the fair dealing and educational exceptions to copyright (Copyright Act, R.S.C., 1985). The unmatched stimuli ranged from 1.84 to 3.71 s in length, with an average of 2.38 s in duration, on average. A total of 60 sentences (see Appendix C) were retrieved from the above sources, with half spoken and half sung recordings of solo voices (no instruments in the sung versions). Sentence text and speaker were not matched in this unmatched set, so no sentences were repeated. Although these stimuli were not matched for overall duration, pitch, etc., they were equated for total RMS amplitude. The acoustic features and derived rhythm metrics are reported for each stimulus set separately in Table 1, and the description and method for extracting each feature is reported in Appendix B.

**Table 1:** Acoustic features extracted for all matched and unmatched stimuli, using Praat-based linguistic metrics, Music Information retrieval metrics from MIR Toolbox, and music-inspired regularity metrics.

		Matched			Unmatched			Speech	Song	*P*	Speech	Song	*P*
Praat-based metrics	F0	138.45 (20.09)	138.15 (11.41)	0.930	158.88 (55.86)	277.53 (75.59)	<0.001
	F0 instability	1.40 (0.50)	0.68 (0.14)	<0.001	1.23 (0.38)	0.97 (0.34)	0.006
	Total duration	2.43 (0.33)	2.49 (0.37)	0.381	2.29 (0.23)	2.48 (0.42)	0.030
	Syllable duration	0.26 (0.04)	0.27 (0.04)	0.196	0.21 (0.04)	0.39 (0.11)	<0.001
	Stressed duration	0.37 (0.08)	0.37 (0.09)	0.771	0.31 (0.12)	0.43 (0.24)	0.020
	Vocalic nPVI	53.61 (14.49)	54.44 (16.35)	0.792	59.66 (16.73)	72.02 (26.37)	0.035
	Consonantal PVI	117.87 (39.83)	108.93 (32.83)	0.233	95.20 (51.39)	184.16 (71.25)	<0.001
	Stress syllable nPVI	51.07 (15.24)	51.88 (13.74)	0.784	51.95 (21.11)	67.59 (32.94)	0.033
	Syllable nPVI	61.96 (15.32)	57.00 (15.16)	0.114	55.39 (14.83)	65.06 (23.23)	0.060
	%V	0.49 (0.07)	0.55 (0.08)	<0.001	0.48 (0.08)	0.66 (0.09)	<0.001
	ΔC	0.08 (0.02)	0.07 (0.02)	0.154	0.07 (0.03)	0.08 (0.04)	0.288
	ΔV	0.07 (0.02)	0.07 (0.02)	0.002	0.06 (0.02)	0.21 (0.10)	<0.001
Music information retrieval	Spectral flux	45.17 (4.42)	38.81 (4.12)	<0.001	107.44 (40.93)	90.44 (20.45)	0.048
	Sub-band flux 1	1.36 (1.37)	1.01 (0.56)	0.107	1.36 (0.67)	1.397 (1.01)	0.880
	Sub-band flux 2	1.36 (1.37)	1.01 (0.56)	0.107	4.85 (3.89)	1.11 (0.37)	<0.001
	Sub-band flux 3	7.13 (2.03)	6.33 (2.42)	0.080	40.58 (32.16)	13.74 (17.82)	<0.001
	Sub-band flux 4	13.27 (3.01)	10.71 (2.47)	<0.001	46.03 (21.90)	33.14 (16.98)	0.014
	Sub-band flux 5	20.56 (4.44)	17.75 (4.21)	0.002	28.92 (8.87)	26.65 (12.85)	0.429
	Sub-band flux 6	12.95 (3.63)	11.28 (3.58)	0.026	19.36 (7.57)	24.93 (15.61)	0.085
	Sub-band flux 7	10.84 (3.59)	10.45 (3.92)	0.614	13.99 (7.15)	20.47 (9.31)	0.004
	Sub-band flux 8	6.56 (2.37)	5.76 (2.25)	0.091	8.21 (4.24)	10.53 (6.28)	0.099
	Sub-band flux 9	2.82 (1.42)	2.10 (0.90)	0.004	7.92 (6.73)	12.33 (9.52)	0.022
	Pulse clarity (Max)	0.22 (0.10)	0.23 (0.10)	0.757	0.23 (0.08)	0.23 (0.09)	0.948
	Pulse clarity (Min)	0.16 (0.06)	0.16 (0.06)	0.571	0.20 (0.06)	0.19 (0.05)	0.603
	Tempo (autocorr)	127.87 (36.19)	132.70 (36.93)	0.524	116.90 (26.70)	110.47 (30.63)	0.390
	Tempo (spectrum)	146.14 (29.10)	144.77 (26.68)	0.812	141.08 (28.74)	126.55 (27.83)	0.054
Music-inspired metrics	Integer multiple	0.35 (0.20)	0.36 (0.20)	0.893	0.37 (0.16)	0.36 (0.27)	0.925
	Asynchrony	0.12 (0.12)	0.11 (0.13)	0.703	0.16 (0.12)	0.16 (0.13)	0.366
	Asynchrony SD	0.12 (0.11)	0.12 (0.12)	0.743	0.14 (0.12)	0.12 (0.12)	0.489
	Signed asynchrony	0.04 (0.14)	0.02 (0.15)	0.444	0.11 (0.19)	0.06 (0.16)	0.324
	Signed SD	0.14 (0.12)	0.13 (0.13)	0.791	0.16 (0.12)	0.14 (0.13)	0.458

Average (st dev) value for spoken and sung exemplars, the *p*-value (uncorrected paired samples *t*-test) characterizes whether the metric differed for speech and song.

### Procedure

The procedure was similar to Experiment 1, except that the stimuli from the unmatched and matched datasets were blocked and rated separately from one another. Participants were asked to wear headphones and complete the surveys in a distraction-free environment. The same order–matched stimulus set, followed by the unmatched stimulus set–was used for all participants so as not to increase variability in ratings across stimulus sets and for maximal comparison to Experiment 1. Prior to each rating task, participants heard a training section with 4 training stimuli that provided examples of spoken and sung utterances that were easy and hard to clap to. Training utterances were spoken and sung by a single male speaker using the text and melody of the familiar children’s song “Twinkle, Twinkle, Little Star” (Taylor and Taylor, 1806), and were labeled as “Song” or “Speech” and “Easy to tap or clap along to” or “Not easy to clap or tap along to.” Easy to tap/clap utterances were sung with a strict metrical pulse or spoken like a poem with a clear prosodic metrical foot alternation. The other stimuli were performed with temporal irregularities including saying words quickly and with irregular pauses between words to disrupt any perception of a beat. Participants could listen to these examples as many times as they wanted and had to listen to all 4 to move forward in the survey. For each stimulus in the rating task, participants rated “How easy would it be to clap or tap to that clip?” with a rating scale of “1 = Not Very Easy” through to “9 = Very Easy.” As before, participants could listen to the clips as many times as they wanted but had to listen at least once to move forward. Participants completed an unrelated task [the SSS test reported in Assaneo et al. (2019)] between the matched and unmatched ratings, but those data are beyond the scope of the current paper and are not reported here. The same two catch (“attention check”) trials were used from Experiment 1 and were randomly incorporated in each block (4 in total). Finally, participants filled out a demographic background questionnaire.

### Results

**Figure 2:**
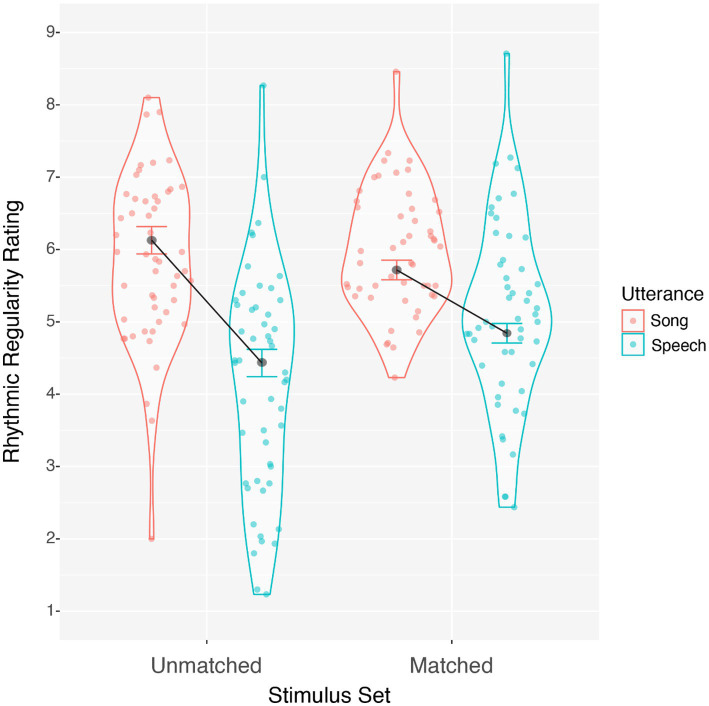
Average rhythmic regularity ratings for song and speech grouped by matched and unmatched stimulus sets, within-subjects standard error (Morrey, 2008).

Rhythmic regularity ratings were averaged separately for spoken and sung utterances in both the matched and unmatched stimulus sets and submitted to a 2 (Utterance: speech, song) by 2 (Stimulus set: matched, unmatched) repeated-measures ANOVA. Song was rated as more rhythmically regular than speech, *F*(1, 50) = 39.490, *p* < 0.001, η_p_^2^ = 0.441, and matched stimuli had higher regularity ratings than unmatched stimuli, *F*(1, 50) = 21.089, *p* < 0.001, η_p_^2^ = 0.297. However, a significant interaction between stimulus set and utterance, *F*(1, 50) = 13.899, *p* < 0.001, η_p_^2^ = 0.218, suggested that the effect of utterance type was larger in the unmatched than the matched set, as illustrated in Figure 2. Simple effects revealed that for matched stimuli, song ratings were higher than speech ratings by 0.874 units on the rating scale, *F*(1, 50) = 20.863, *p* < 0.001, η^2^ = 0.294. For the unmatched stimuli, song ratings were higher than speech by 1.696 units on the rating scale, *F*(1, 50) = 40.338, *p* < 0.001, η^2^ = 0.447. Overall, song was consistently rated as more rhythmically regular than speech, but this difference was larger for unmatched compared to matched utterances. These findings indicate that a clear definition of rhythmic regularity allows listeners to be sensitive to rhythmic regularity as a distinguishing feature between music and language. Participants were sensitive to differences in rhythmic regularity in acoustically constrained settings as well, when features that are typically correlated with regularity, like tempo, are held constant across spoken and sung exemplars.

### Correlating rhythmic measures with subjective ratings

To examine which acoustic features best predicted listeners’ rhythmic regularity ratings, we included features that were correlated with regularity ratings in a linear mixed effects model. First, we performed first order correlations among all the extracted metrics (see Method and Supplementary Table S4) despite redundancy across rhythmic measures. Unmatched spoken and sung utterances differed greatly in the number of syllables (fewer for song than speech), which affected several other metrics including average syllable duration and metrics related to syllable or vocalic/consonant onsets. We performed separate first order correlations for matched and unmatched stimulus sets to ensure that features correlated in one set but not another due to syllable number had the opportunity to be entered into the model (see Supplementary Table S4). Several first order correlation features were highly correlated with other predictors, such that F0, syllable duration, stressed interval, %V, consonantal PVI, and ΔV were all correlated with one another (all *rs* > 0.3, see Supplementary Table S5). To reduce multicollinearity, the feature that was most highly correlated with rhythmic regularity was entered for model testing (i.e., average syllable duration, see Supplementary Table S4). Spectral flux was correlated with each sub-band flux metric. Total spectral flux was chosen for model testing over any sub-band measure because overall flux correlated consistently with rhythmic regularity in each stimulus set, while sub-band flux correlations were present or absent depending on the stimulus set. The final features entered into the model were F0 instability, total duration, average syllable duration, and spectral flux (but see Supplementary Table S6 for additional analyses using consonantal PVI and %V instead of syllable duration). All measures were mean-centered and any measures with kurtosis or skewness (+/−3) were log-transformed and mean-centered before being entered into the model.

**Figure 3:**
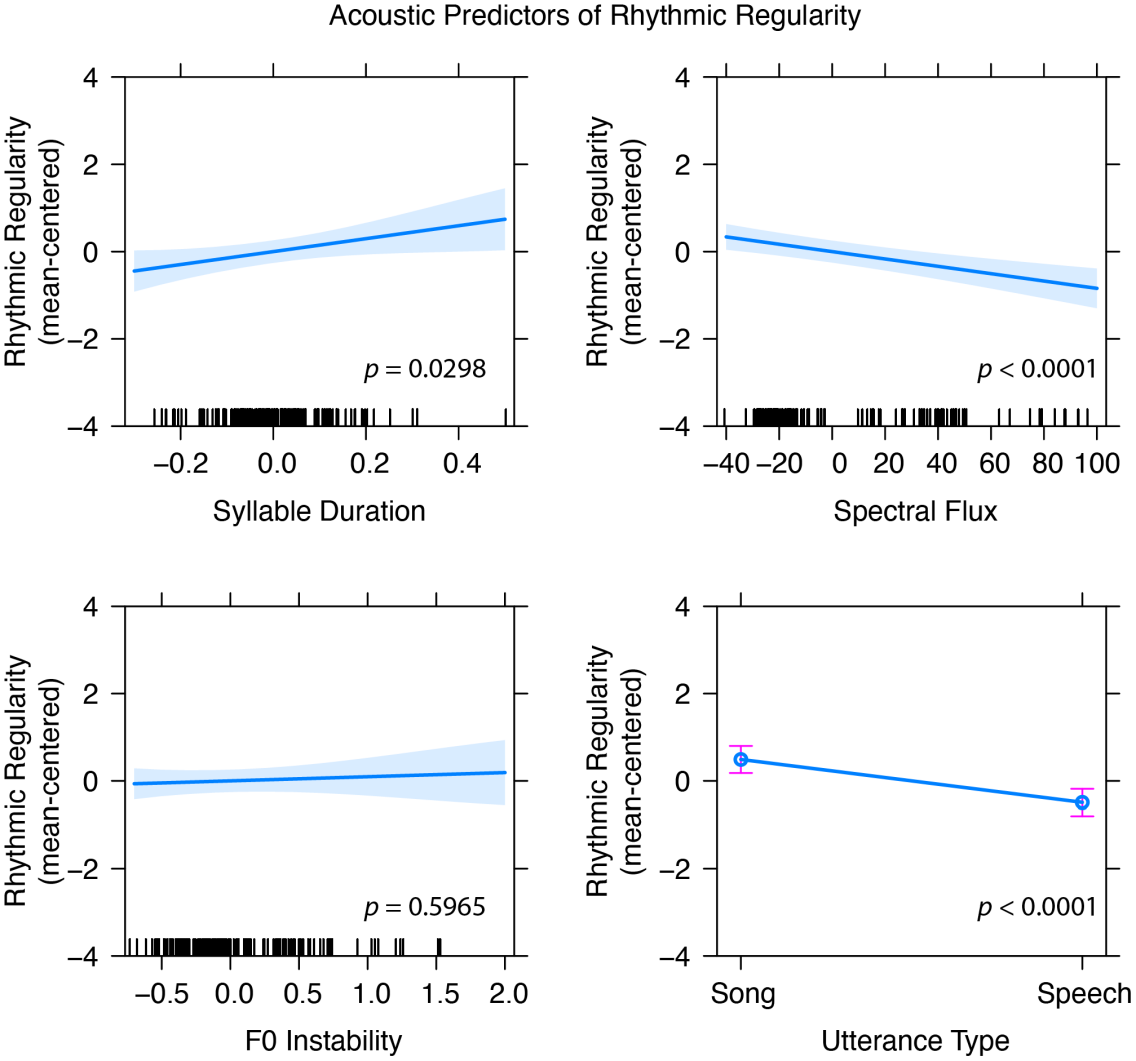
Model 4 indicates that syllable duration and spectral flux are significant predictors of perceived rhythmic regularity even after accounting for utterance type (speech vs. song), but F0 instability, which was significant in models without utterance type as a factor, is no longer a significant predictor of perceived regularity. Error bars for utterance type and shaded error regions represent standard error calculated using the Kenward-Roger coefficient covariance matrix (Effect package, R).

Participant ID and Stimulus ID were entered as random effects, with 1 spectral and 3 temporal features added as fixed effects. These fixed effects significantly improved the fit of the basic model (see Table 2, Model 1), but duration did not uniquely contribute to the model. After removing duration, Model 2 accounted for a significant amount of variance compared to the random effects model and Model 1 did not account for more variance than the Model 2 (*p* = 0.743). Model 3 included syllable count to ensure that predictors were robust to the small number of syllables present in sung utterances from the unmatched condition. Syllable count did not significantly improve fit compared to Model 2 (see Table 2, Model 3), and did not change the significance of average syllable duration. Finally, Model 4 examined whether the acoustic features from Model 2 would remain significant even after adding speech and song labels into the model (utterance type). F0 Instability was no longer significant in this final model, presumably because F0 stability was more predictive of speech-song differences than regularity within stimulus classes. Thus, in addition to songs having greater rhythmic regularity than speech, stimuli with longer syllable durations and less spectral flux were rated as more rhythmically regular (Figure 3).

**Table 2:** LME models predicting rhythmic regularity.

Model	Variable	Estimate	t-value	*P*
Model 1:	Duration	0.074	0.323	0.7469	Syllable duration	**3.270**	**4.668**	**<0.0001**	spectral flux	**−0.008**	**−3.521**	**0.0006**	F0 instability	**−0.467**	**−2.980**	**0.0034**
***X***^***2***^**(8, *N* = 7,954) = 61.254, *p* < 0.001, AIC = 32,857** (*compared to random intercept model*)				
Model 2:	Syllable duration	**3.372**	**5.412**	**<0.0001**	Spectral flux	**−0.008**	**−3.625**	**0.0004**	F0 instability	**−0.466**	**−2.986**	**0.0033**
***X***^***2***^**(7, *N* = 7,954) = 61.1468, *p* < 0.001, AIC = 32,855** (*compared to random intercept model*)				
Model 3:	Syllable count	−0.018	−0.299	0.7718	Syllable duration	**3.121**	**2.985**	**0.0033**	Spectral flux	**−0.008**	**−3.546**	**0.0005**	F0 instability	**−0.467**	**−2.985**	**0.0033**
***X***^***2***^**(8, *N* = 7,954) = 0.0921, *p* = 0.7615, AIC = 32,857** (*compared to model 2*)				
Model 4:	Utterance type (speech)	**−0.980**	**−5.297**	**<0.0001**	Syllable duration	**1.483**	**2.194**	**0.0298**	Spectral flux	**−0.008**	**−4.363**	**<0.0001**	F0 instability	−0.095	−0.531	0.5965
***X***^***2***^**(8, *N* = 7,954) = 26.464, *p* < 0.0001, AIC = 32,830** (*compared to model 2*)				

Model 4 is the best fitting model, with syllable duration and spectral flux predicting rhythmic regularity even after accounting for stimulus type (speech vs. song). Model 3 and Model 4 illustrate syllable duration and spectral flux are robust predictors of rhythmic regularity even after accounting for the number of syllables in an utterance and stimulus type. Bold variables indicate significant predictors to the model.

## Interim discussion

A major goal of Experiment 2 was to standardize participants’ interpretation of rhythmic regularity by providing a concrete definition centered on ease of clapping or tapping along with the stimulus. With this definition, rhythmic regularity ratings were significantly higher for sung than spoken utterances. Experiment 2 also expanded on the acoustically matched stimulus set from Experiment 1 by including an additional unmatched stimulus set more representative of speech and song in everyday settings. Participants rated song as more rhythmically regular than speech for both sets, but the difference was larger for the acoustically unmatched than the matched set. Naturally recorded utterances may emphasize the differences in regularity between song and speech compared to recordings that equate tempo, pitch contour, and average pitch between speech and song. However, regularity differences are apparent even in carefully acoustically matched stimulus sets, suggesting that regularity helps differentiate speech and song. Finally, we estimated which acoustic features across both stimulus sets were most predictive of regularity ratings. Although the type of stimulus (speech or song) was a significant predictor of regularity, longer syllable durations and less spectral flux also predicted higher rhythmic regularity ratings.

## General discussion

The goal of this work was to obtain a subjective metric of rhythmic regularity—an equally-spaced, repeating pulse—and examine acoustic features that predict participants’ ratings of regularity. Experiment 1 illustrated that the term rhythmic regularity was interpreted differently across participants, leading to different patterns of regularity across spoken and sung exemplars. Experiment 2 operationalized the definition of rhythmic regularity by asking how easy it would be to tap or clap to the stimulus. With this definition, participants rated song as more regular–or easier to clap or tap to–than speech in both acoustically matched and acoustically unmatched stimulus sets. Subjective regularity ratings were significantly affected by acoustic features of syllable duration and spectral flux, with longer durations and less flux related to higher regularity ratings. These results add to the literature by (1) highlighting the salience of rhythmic regularity as a differentiator of speech and song (Patel and Daniele, 2003; Patel et al., 2005; Vanden Bosch der Nederlanden et al., 2022b) and (2) adding to the growing literature on spectral flux as a salient acoustic feature in listeners’ perceptual processing of sound (Weineck et al., 2022).

Spectral flux is a metric of the distance between successive frames, or moments in time, in the frequency spectrum, with larger values indicating large changes in the spectrum from moment to moment (Alluri and Toiviainen, 2010). It logically follows that song should have less spectral flux since notes are held longer (i.e., greater proportion of the utterance is vocalic) than in speech, creating fewer changes in the spectrum on a moment-to-moment basis. The metrical framework of sung utterances may also make for fewer sudden and more evenly spaced changes in the spectrum compared to speech. Spectral flux has been described as an acoustic correlate of the beat in music, but with greater spectral flux indicating greater beat salience (Burger et al., 2013). These authors extracted spectral flux from low and high frequency bands in the spectrum corresponding to the kick drum, hi-hat, and cymbal. For this reason, large amounts of spectral flux in these bands acted as a proxy for rhythmic information from these instruments. These stimulus-specific differences help to explain the seeming paradox of greater spectral flux predicting more beat salience in music, while greater spectral flux predicts less rhythmic regularity when comparing speech to song.

Our results elucidate what features participants use to provide regularity ratings when comparing speech and song, but these features alone are unlikely to capture the presence of a beat or the integer multiple relatedness of sounds snapping to the metrical grid across a wide range of environmental stimuli. We attempted to account for listeners’ subjective regularity ratings using several music- and language-inspired metrics of regularity. In particular, the proportion of intervals per sentence that were related by integer multiples (Roeske et al., 2020) was not correlated with regularity ratings. It may be that our sentence-level approach is too coarse a metric and behavioral responses like tapping or continuous regularity ratings could shed light on which features participants relied on at particular moments in time to feel a beat (similar to Rathcke et al., 2021). The consistency with which those moments align with inter-onset-interval or stimulus features could provide a path forward for creating novel metrics to characterize regularity differences in speech and song. Another set of metrics used for this study (Asynchrony, Signed Asynchrony and their variability) was inspired by the clock timing work from Povel and Essens (1985) (similar to Norton and Scharff, 2016 for birdsong). However, this metric also failed to provide any relationship to subjective regularity and may also require input from the p-center-related literature (e.g., Rathcke et al., 2021) to determine the correct beat locations and onset times used to develop the underlying “clock” for speech and song. Onset intervals related to vocalic or other salient features of the stimulus may be more fruitful than the reliance on linguistic onsets used here. Finally, music information retrieval metrics like pulse clarity and stimulus-extracted tempo had no relationship to rhythmic regularity in speech and song, suggesting that these feature extraction methods are perhaps better suited for use with multi-instrument (e.g., vocals and instrumentation) excerpts of musical pieces rather than vocal sung and spoken utterances.

Linguistic measures, including measures that have previously been used to relate speech and music to one another, such as nPVI, also did not explain additional variance in rhythmic regularity beyond average syllable duration (see Supplementary Table S6). Vocalic nPVI was originally developed to capture the vowel reduction (i.e., change in vowel quality to a “schwa” and shortened duration of vowel length) that happens in many of the so-called “stress-timed” languages (Grabe and Low, 2002; Patel et al., 2005; Cummins, 2012). This measure is not best at capturing rhythmic variability, but rather contrastiveness between pairs of syllables. Indeed, our calculations indicated that music often had more contrastiveness than speech (see Table 1, Unmatched stimuli), which is likely due to large integer-related duration differences like quarter notes to half or whole notes that speech does not employ. Comparisons of previous work from separate studies suggested that nPVIs were much higher for speech (in the 50–70 range) than instrumental music (in the 30–40 range; Patel and Daniele, 2003; Hannon et al., 2016), but these studies used musical notation to estimate nPVI durations instead of actual recordings. Studies that have used acoustic segmentation of speech and song have illustrated more comparable nPVI values (Vanden Bosch der Nederlanden et al., 2022a,b). Thus, it is not surprising that this metric did not uniquely predict rhythmic regularity for spoken compared to sung stimuli.

Despite the ease with which humans pick up on regularity in speech, song, and environmental sounds, easily extractable acoustic features that characterize those subjective reports remain elusive. Our study confirms that participants hear more rhythmic regularity in sung compared to spoken utterances, providing concrete metrics for how best to obtain participant’s subjective regularity ratings. The findings from this study also add to the literature by characterizing that regularity is easier to detect–or more likely to be present–when syllables are longer, and when there is less moment-to-moment fluctuation in the spectrum. Future work should build on these results to develop more continuous and fine-grained metrics for quantifying rhythmic regularity from the acoustic signal. There is growing evidence that rhythmic regularity is an important signal for attention, perception, development, and movement (Grahn and Brett, 2007; Gordon et al., 2014; Bedoin et al., 2016; Trainor et al., 2018; Aman et al., 2021; Lense et al., 2021) in humans, and is present in a range of human and non-human primate communicative vocalizations (Roeske et al., 2020; De Gregorio et al., 2021), as well as many environmental sounds (Gygi et al., 2004). Indeed, the perception of rhythmic regularity is key to how both human and non-human animals (e.g., cockatoos, sea lions) align their movements to a beat (Fitch, 2013). A greater understanding of what acoustic features humans rely on to perceive regularity and extract an underlying pulse in communicative signals like speech and song will contribute to theories of evolutionary origins of beat processing (e.g., are the features humans use to find a beat the same or different from animals?) and theories about perceptual biases toward regularity in everyday soundscapes.

One potential limitation of the current study is the use of lyrics in both the music and language domains. We wanted to use speech and song because they exemplify the acoustic and structural differences between domains (Vanden Bosch der Nederlanden et al., 2022a,b), while maintaining the ability to control for timbral, semantic, and other temporal or spectral acoustic features. It will be important to characterize the role linguistic content plays in the perception of rhythmic regularity in song. For instance, is song without words perceived as more strictly regular that song with words given that note durations are less dictated by word length or stress? If so, then are instrumental melodies perceived as more rhythmically regular than songs without words? Or does linguistic or semantic content help to bolster temporal prediction for what type of note and/or word will come next? Similarly, would speech without semantic content (e.g., low-pass filtered) be perceived as more or less regular than semantic speech? This and future work will help shed light on the temporal features that distinguish speech and song and, more broadly, the domains of music and language.

The current findings add to the literature on rhythm in music and language by providing a concrete subjective metric of rhythmic regularity that reliably differs between speech and song across stimulus sets. The metric is simple to understand and can be used to characterize the perception of rhythmic regularity across developmental populations, in individuals with little or no musical training, and in a range of stimulus sets beyond music and language (e.g., bird song). Our findings are important for characterizing the inherent differences in music and language that (1) may be important for learning to differentiate musical and linguistic communication early in development (Vanden Bosch der Nederlanden et al., 2022a,b) and (2) underlie many of the perceptual advantages ascribed to music over language. For instance, cross-culturally humans prefer simple integer ratios in music (Jacoby and McDermott, 2017) and remember these musical rhythms better than syncopated rhythms that disrupt the occurrence of events on a beat (Fitch and Rosenfeld, 2007). Future work comparing the prominence of features in speech compared to song could address the divergence of musical and linguistic communication in humans. For instance, does the preservation of rhythmic regularity in music come at a cost to the transmission of quick messages meant to transact information? Is strict isochrony better for promoting verbatim memory of information occurring on, but not off the beat (Jones et al., 1981; Large, 2008; Helfrich et al., 2018) while vague periodicity without strict isochrony (as in speech) is better for encoding the gist of a message? Answering seemingly simple questions like how humans perceive differences in rhythmic regularity in speech and song, has the potential to address several important areas of psychology related to human communicative development, origins of music and language, cross-species comparisons, and perceptual biases toward regularity in everyday scenes.

## Data availability statement

The datasets presented in this study can be found in online repositories. The names of the repository/repositories and accession number(s) can be found at: https://osf.io/hnw5t/.

## Ethics statement

The studies involving human participants were reviewed and approved by the University of Western Ontario Ethics Board. The patients/participants provided their written informed consent to participate in this study.

## Author contributions

CY, JG, and CV designed the experiments. CY and CV recruited the participants and performed the data analysis. CV and AC extracted acoustic features and manually segmented stimuli. CY wrote the first draft. CV provided subsequent drafts. All authors contributed to the article and approved the submitted version.

## Funding

This work was supported by NSERC RGPIN-2016-05834 awarded to JG and NSERC RGPIN-2022-04413 and DGECR-2022-00294 awarded to CV.

## Conflict of interest

The authors declare that the research was conducted in the absence of any commercial or financial relationships that could be construed as a potential conflict of interest.

## Publisher’s note

All claims expressed in this article are solely those of the authors and do not necessarily represent those of their affiliated organizations, or those of the publisher, the editors and the reviewers. Any product that may be evaluated in this article, or claim that may be made by its manufacturer, is not guaranteed or endorsed by the publisher.
