# Supplementary material for: Perceived rhythmic regularity is greater for song than speech: examining acoustic correlates of rhythmic regularity in speech and song
Source: Front Psychol. 2023 May 26;14:1167003. doi: 10.3389/fpsyg.2023.1167003 (PMC10250601; doi:10.3389/fpsyg.2023.1167003)
Supplement: Supplementary file 1 [file Data_Sheet_1.PDF]

Supplemental Materials

Experiment 1 – Participant Demographics

Table S1. Demographics of participants in Experiment 1

**Variable**	**Value**
Bilingual (self-identified)	*N*=11
English From Birth	*N*=22
Age began English	5.11 (range: 2 – 10) years
Languages	Chinese (9), Arabic (1), Russian (1)
Music Training	*N*=11
Age began	8.64 (2.72) years
Duration training	7.64 (3.80) years
Racial Background	(not mutually exclusive)
White	N = 15
Chinese	N = 12
Asian Indian	N = 4
Southeast Asian	N = 2
Middle Eastern	N = 2

Experiment 1 – Thematic coding of rhythmic regularity definitions

First, subjective definition of rhythmic regularity were coded by two of the authors without looking at the direction of the effect (regularity ratings for speech and song). Themes helped to provide explicit criteria for inclusion in beat-based and other rhythmic regularity definition groups. Based on the themes, the criteria for beat-based definitions included the explicit mention of beat or meter and/or the importance of rhythmic consistency, prosody-based talked more about linguistic stress and normalness of conversation, whereas unclear definitions did not fall in either beat or prosody-based definitions.

Table S2. Definition and count of endorsed themes grouped by definition across all participants.

Definition	Beat Meter	Rhythmic Consistency	Music Training	Normal Tempo/Flow	Normal For Conversation	Word Stress	Pitch Prosody	Rhyme	Other
Beat	5	8	1	5	0	1	0	0	4
Prosody	0	1	0	4	6	4	5	2	5
Unclear	0	1	1	4	2	0	3	0	10

Note: The sum of each row does not add up to number of participants in each group (12, 11, and 10, respectively), but instead the number of themes endorsed per group. This table illustrates that beat-based definitions were based on mention of beat, meter, and/or rhythmic consistency. Prosody-based definitions were based on mentions of word stress and prosodic pitch, but most often on how normal the utterance sounded for a conversation. Finally, unclear definitions were mostly based on “Other” codes which did not relate to acoustic features. To see a full table of definitions, themes, codes, see uploaded data file on OSF.

Beat-based definitions

- That means I need to determine whether each beat is formed into a particular pattern.
- if the sound has a very noticeable rhythm or if it is off beat and all over the place
- The clips have a sense of rhythm. The whole sentence has a tempo like 4/4 tempo or 2/4 tempo. And it means I can beat the rhythm.
- How well the phrase fit in with a standard rhythmic meter, e.g. 4/4 time or 6/8 time. To determine this, considered mainly spacing between syllables but also which syllables were emphasized in enunciation.
- Whether the person sounded like they had rhythm. I guess did the voice and sound match someone who was musically gifted
- rate them based on how regular the rhythmic pattern was
- How consistent was the rhythm
- whether it normal/ consistant rhytm to it
- High rhythmic regularity was interpreted as a clip that had a smooth tempo/consistent beat.
- A steady pace or a repeated rhythm
- I would try to ignore the musicality of the notes and only tap out the syllables to see if they were spaced out evenly or not

Prosody-based definitions

- To me, it meant the pitch and tone of the voice. I tried not to pay attention to what the speaker was saying, but the emphasis they placed on different words.
- A combination of flow, intonation and expression relative to how the words sounded.
- How well the words flowed together; some words sound clunky and make the sentence uneven. Intonation and accent can make the words sound better or worse. Rhymes and similar word sounds (alliteration) have the highest rhythmic regularity.
- To me, rhythmic regularity meant the way the person was speaking. Something is classified as regular rhythm as you talk normal as if giving direction. Something is not regular if there is a rhythm such as a pattern or musical background to it.
- Rhythmic regularity to me means saying the words and phrases with the proper rhythm. By this I mean holding syllables and pauses for the proper length of time.
- any phrases that sounded as though they could be spoken in a normal conversation and heard without raising question or concern for the other persons speech abilities.
- Rhythmic regularity meant how regular the phrases sounded if they were to be spoken in normal conversations. For instance, a phrase that sounded quite abrupt and was said with an odd tune was rated less and a phrase spoken in a normal monotone fashion was rated high (very regular). Additionally, phrases spoken with some tune and rhythm that sounded good was rated moderately high.
- If it sounded the same as if someone was speaking the phrase normally
- To me, rhythmic regularity meant how common that particular flow for a phrase is used in the real world.
- how normal they sound to me, how often I heard them from my daily life.
- The way they rhyme words and talk or sing in the same tune

Unclear definitions

- If it made me feel annoyed or I disliked it I considered it rhythmicly irregular
- In my mind, rhythmic regularity is based on the audio that was most regular or familiar to me. If the phrase was said in a common tune, I would say it is more rhythmically regular to me. Whereas, if the phrase or the tune was something I am not familiar with, it is less regular to me.
- How consistent it was. Like when the pitches changed and the lengths between each word.
- This means that the sentence sounds like normal talking/singing and the rhythm is not weird
- It meant that the audio clips had to sound sound "pleasing" and flow well, and have a "repeated" and "regular" pattern.
- It means that I need to listen to a voice to objectively judge the rhythm of this sentence
- To test my sensitivity to sound and rhythmic
- How smooth and easily repeatable the audio was. I think that is what rhythm means to me, I have practically no background in music.
- It was either measuring how much they were singing or how monotone they were... one of the two.
- I considered "rhythmically regular" to be that the speed of the audio sounds normal, like a normal conversation or in music.

Experiment 2: Participant Demographics

Table S3. Demographics of participants in Experiment 2

Variable	Value
Bilingual (self-identified)	*N*=16
English From Birth	*N*=36
Age began English	6.30 (range: 2 – 12) years
Languages	Chinese (6), Farsi (3), Arabic (2), Vietnamese (2), One each: Indonesian, Tagalog, Korean
Music Training	*N*=12
Age began	9.30 (SD = 2.83) years
Duration training	5.47 (SD = 3.14) years
Racial Background	(not mutually exclusive)
White	N=25
Chinese	N = 11
Southeast Asian	N = 5
Asian Indian	N = 5
Middle Eastern	N = 4
African	N = 4
South Asian	N = 2
South American	N = 2

Experiment 2

Table S4. First order correlations between all features and rhythmic regularity ratings

	**Matched - Ratings**	**Unmatched - Ratings**
F0	0.082 (0.425)	0.514 (<.001)***
F0 Instability	-0.350 (<.001)***	-0.289 (0.025)*
Total duration	0.237 (0.020)*	0.273 (0.035)*
Syllable duration	0.107 (0.301)	0.779 (<.001)***
Stressed interval duration	0.170 (0.098)	0.451 (<.001)***
Stressed interval variability	0.148 (0.151)	0.079 (0.553)
Stressed Syllable nPVI	-0.017 (0.872)	0.166 (0.204)
Syllable nPVI	-0.114 (0.270)	0.166 (0.244)
Tempo (spectrum)	-0.110 (0.284)	-0.199 (0.127)
Tempo (autocorrelation)	-0.067 (0.516)	-0.191 (0.143)
Tempo (spectral flux)	0.095 (0.355)	0.057 (0.663)
Maximum pulse clarity	0.110 (0.287)	0.120 (0.362)
Minimum pulse clarity	0.001 (0.989)	-0.036 (-.786)
Spectral flux	-0.263 (0.010)**	-0.412 (<.001)***
Spectral flux, sub-band 1	-0.094 (0.362)	-0.154 (0.240)
Spectral flux, sub-band 2	-0.094 (0.362)	-0.600 (<.001)***
Spectral flux, sub-band 3	0.109 (0.288)	-0.635 (<.001)***
Spectral flux, sub-band 4	-0.215 (0.036)	-0.321 (0.012)*
Spectral flux, sub-band 5	0.018 (0.866)	-0.121 (0.356)
Spectral flux, sub-band 6	-0.276 (0.007) **	0.174 (0.184)
Spectral flux, sub-band 7	-0.116 (0.262)	0.319 (0.013)*
Spectral flux, sub-band 8	-0.361 (<.001)***	0.160 (0.222)
Spectral flux, sub-band 9	-0.140 (0.172)	0.073 (0.580)

Integer multiples	-0.135 (0.190)	-0.083 (0.529)
Asynchrony	-0.017 (0.870)	-0.067 (0.609)
Asynchrony variability	0.013 (0.902)	-0.064 (0.627)
Signed asynchrony	-0.064 (0.539)	-0.099 (0.451)
Signed variability	-0.014 (0.894)	-0.067 (0.612)
Vocalic nPVI	-0.059 (0.568)	0.169 (0.197)
Consonantal PVI	0.083 (0.420)	0.672 (<.001)***
ΔC	0.011 (0.919)	0.202 (0.121)
ΔV	0.065 (0.532)	0.587 (<.001)***
%V	0.124 (0.229)	0.550 (<.001)***
Number of Syllables	0.102 (0.323)	-0.755 (<.001)***

Note: Highlighted variables are highly correlated with one another in Table S5.

Table S5

First Order Correlations for Variables Correlated with Subjective Rhythmic Regularity

Variable		1. F0	2 Stability	3 Tot Dur	4 Syll Dur	5 Stress Dur	6 SF	7 %V	8 cPVI
2. F0 Stability	r	-0.051	—						
	p	0.527	—						
3. Total Duration	r	0.029	-0.102	—					
	p	0.715	0.205	—					
4. Syllable Duration	r	0.433 ***	-0.147	0.452 ***	—				
	p	< .001	0.067	< .001	—				
5. Stressed Interval Duration	r	0.070	-0.156	0.367 ***	0.450 ***	—			
	p	0.386	0.052	< .001	< .001	—			
6. Spectral Flux	r	0.477 ***	0.259 **	-0.221 **	-0.066	-0.197 *	—		
	p	< .001	0.001	0.006	0.415	0.014	—		
7. %V	r	0.523 ***	-0.088	0.223 **	0.514 ***	0.217 **	0.174 *	—	
	p	< .001	0.274	0.005	< .001	0.006	0.030	—	
8. Consonantal PVI	r	0.302 ***	-0.101	0.088	0.658 ***	0.260 **	-0.033	0.164 *	—
	p	< .001	0.210	0.277	< .001	0.001	0.679	0.041	—
9. ΔV	r	0.531 ***	-0.107	0.267 ***	0.743 ***	0.104	0.182 *	0.621 ***	0.495 ***
	p	< .001	0.182	< .001	< .001	0.198	0.023	< .001	< .001

* p < .05, ** p < .01, *** p < .001

Table S6. Supplemental Regression with Language-Based Metrics instead of Syllable Duration

**Model**	**Variable**	**Estimate**	**t-value**	***p***
Model 1:	Duration	0.345	1.639	0.1032
	**%V**	**3.270**	**4.668**	**0.0021**
	**Consonantal PVI**	**1.342**	**3.459**	**0.0007**
	**Spectral Flux**	**-0.009**	**-4.103**	**<0.0001**
	**F0 Instability**	**-0.439**	**-2.812**	**0.0056**
**X^2(5, *N*=7954) = 64.530, *p<.001*, AIC = 32855** (*compared to random intercept model*)				
Model 2:	**%V**	**2.647**	**3.678**	**0.0003**
	**Consonantal PVI**	**1.347**	**3.902**	**0.0007**
	**Spectral Flux**	**-0.010**	**-4.640**	**<0.0001**
	**F0 Instability**	**-0.440**	**-2.805**	**0.0057**
**X^2(4, *N*=7954) = 61.773, *p<.001*, AIC = 32856** (*compared to random intercept model*)				
Model 3**:**	Syllable Count	-0.046	-0.845	0.3994
	**%V**	**2.325**	**2.851**	**0.0050**
	**Consonantal PVI**	**1.052**	**2.005**	**0.0468**
	**Spectral Flux**	**-0.009**	**-4.228**	**<0.0001**
	**F0 Instability**	**-0.445**	**-2.828**	**0.0053**
**X^2(1, *N*=7954) = 0.7375, *p*=0.391, AIC = 32857** (*compared to model 2*)				
Model 4**:**	**Utterance Type (speech)**	**-1.068**	**-5.364**	**<0.0001**
	%V	0.146	0.181	0.8568
	**Consonantal PVI**	**0.913**	**2.484**	**0.0141**
	**Spectral Flux**	**-0.009**	**-4.365**	**<0.0001**
	F0 Instability	0.140	0.774	0.4403
**X^2(1, *N*=7954) = 27.247, *p*<0.0001, AIC = 32831** (*compared to model 2*)				

Supplemental analyses in Table S6 illustrate the same pattern of findings using language-based metrics (i.e., %V and consonantal PVI) of rhythmic regularity instead of the simple metric of syllable duration. The final supplemental model suggested that utterance type (speech vs. song), spectral flux, and consonantal PVI predicted regularity ratings, but %V and F0 Instability did not. Syllable count was also included in supplemental Model 3 and similarly showed that it did not account for the effects of each predictor variable. Results in the main text are displayed using syllable duration models instead of the language-based metrics of %V and consonantal PVI because models showed similar results and the fit of the language-based metrics did not differ from syllable duration despite having more variables to explain variance, $X^2(1, N=7954) = 1.409$, $p=0.2352$, AIC (syllable duration) = 32830; AIC (%V + c PVI) = 32831.
