# Supplementary material for: Perceived rhythmic regularity is greater for song than speech: examining acoustic correlates of rhythmic regularity in speech and song
Source: Front Psychol. 2023 May 26;14:1167003. doi: 10.3389/fpsyg.2023.1167003 (PMC10250601; doi:10.3389/fpsyg.2023.1167003)
Supplement: Supplementary file 2 [file Data_Sheet_2.docx]

**Appendix A - Matched Stimuli**

1. Hop over the fence and plunge in

1a. Rice is often served in round bowls

2. Glue the sheet to the dark blue background

2a. Write at once or you may forget it

3. It’s easy to tell the depth of the well

3a. It’s hard to erase the blue or red ink

4. Yell and clap as the curtain slides back

4a. Shape the clay gently into block form

5. Pour the stew from the pot into the plate

5a. Take the matchstick and strike against your shoe

6. The wide road shimmered in the hot sun

6a. The facts don’t always show who is right

7. Madam, this is the best brand of corn

7a. Women form less than half of the group

8. The boy was there when the sun rose

8a. These thistles bend in a high wind

9. Help the woman get back to her feet

9a. All sat frozen and watched on the screen

10. Press the pants and sew the button on the vest

10a. Footprints show the path he took up to the beach

11. The wagon moved on well-oiled wheels

11a. He crawled with care along the ledge

12. The paper box is full of thumbtacks

12a. A rod is used to catch big salmon

13. Both brothers wear the same size

13a. Two blue fish swam in the tank

14. A king ruled the state in the early days

14a. The bloom of the roses lasts a few days

15. When you hear the bell, come quickly

15a. Slide the bill between the two leaves

16. Mend the coat before you go out

16a. Drive the screw straight into the wood

17. Use a pencil to write the first draft

17a. Prod the old mule with a crooked stick

18. The clock struck to mark the third period

18a. The wood stand is much too high for the couch

19. The least ran out in sixteen weeks

19a. Their eyelids droop for want of sleep

20. We frown when events take a bad turn

20a. The room was crowded with a wild mob

21. Sunday’s the best part of the week

21a. He broke a new shoelace that day

22. To make pure ice, you freeze water

22a. The black trunk fell from the landing

23. They are men who walk in the middle of the road

23a. A round hole was drilled through the thin, thin board

24. The old pan was covered with hard fudge

24a. The gold ring fits only a pierced ear

**Appendix B - Questions**

1. How rhythmically regular did this audio clip sound to you?
2. How easy would it be to tap or clap along to that clip?

**Appendix C - Unmatched Stimuli**

Spoken

1. I reached into the folder one more time
2. The lights were dimmed to a romantic glow
3. His brothers must now identify it
4. The cold passed reluctantly from the earth
5. She had had certain ways of expression
6. The mice sniff and scurry
7. Which they believed would make them feel happy
8. She would have declared herself happy
9. And that was enough to speed up her heart
10. The sandwich was still crumbs in my throat
11. They wanted a dime for pop on the train
12. It didn’t matter so much when you were a little girl
13. And her ball bounded across the room
14. It is a way I have of driving off the spleen
15. Look at the crowds of water-gazers there
16. The loss of smell that you experienced
17. But the tank dropped off closer to Earth
18. Perhaps that’s why the term fell out of favour
19. Our atmosphere protects us from a lot of them
20. This is more of a job than they might think it is
21. Printers thirty years ago were also super annoying
22. I also put a little bit of sea salt in the water
23. Now, the neurons there don’t know what to do
24. It makes sense that it’s not that enjoyable
25. Without the actual knowledge and technology
26. They were so sweet and they were just getting tattoos
27. If you write about memories from your childhood, they’re much richer
28. That’s all I have to say about perspective
29. Do you want to be comfortable dealing with those feelings
30. As long as you can take a deep breath in and out

Sung

1. A world unforgiving
2. I got this look on my face
3. It came without a warning
4. Oh mama how we need you
5. Love, love, love, I love you
6. You can’t just run away
7. We can dance all night
8. Sometimes we fight
9. You had me believing
10. Why did I fall for you
11. I’m sick of waiting for you
12. See you on the other side
13. I love the way you move
14. Hello my friend
15. I get you back
16. I’m flying on my own
17. You don’t even know me
18. You’re in my arms now girl
19. Ever changing life
20. I know the season’s right for change
21. Where will my heart come back to life
22. Do you want to go out with me
23. Everybody’s falling apart
24. This isn’t what I’m about
25. How’d you do it, baby
26. Feel ‘em as clearly
27. I feel like a failure
28. I can’t wait to see you ’gain
29. That I’m just another fool
30. The people thought a coin was hidden there

Appendix D: Acoustic Metrics

*Praat-based:*

**F0** - average fundamental frequency calculated over the whole utterance using the pitch tier automatically generated using the plugin MomelIntsint which finds optimized minimum and maximum F0 ranges to prevent octave transposition errors common in Praat. Spurious pitch moments were deleted when they occurred during fricatives, like sibilant /s/, and were determined by playing the hummed version in Praat and noting if the pitch contour deviated significantly from what the listener experienced in the raw audio file.

**F0 Instability** - standard deviation in semitones of the F0 (from pitch tier) of each syllable averaged across all syllables in an utterance (larger values indicate less stable pitch tiers).

**Total Duration** -Duration in milliseconds from the onset of the first syllable to the offset of last syllable.

**Syllable Duration** - average duration of all syllables in an utterance calculated from duration of each syllable (offset minus onset in milliseconds).

**Stressed Interval Duration** - average onset to onset duration between stressed syllables in an utterance. Stressed syllables were identified using the CMU Pronouncing Dictionary.

**Vocalic Normalized Pairwise Variability Index (vocalic nPVI)** - a standard measure of speech rhythm between voiced onsets (vowels) calculated according to the formula provided by Grabe and Low (2002). Syllable, vocalic, and intervocalic intervals were marked by hand by the experimenter using Peterson and Lehiste’s (1982) guidelines for phonemic segmentation. Similar segments (i.e., two vocalic segments in a row crossing word boundaries) were counted as a single interval for these analyses, ignoring word boundaries as done in Grabe & Low (2002).

**Consonantal raw Pairwise Variability Index (consonantal PVI) -** calculated in the same manner as vocalic nPVI above but using consonant (intervocalic) intervals. This metric was not normalized for speech rate in keeping with best practices reported from Grabe & Low (2002).

**Stressed Syllable nPVI** - calculated as previous nPVI values, but using stressed syllable onset durations and preserving word boundaries (i.e., two stressed syllables in a row were counted as separate durations). Normalized for syllable rate.

**Syllable nPVI** - calculated as previous nPVI values, but using syllable onset durations and preserving word boundaries (i.e., two syllables in a row were counted as separate durations). Normalized for syllable rate.

**Percent V** - percent of the utterance made up of vocalic segments (see Grabe & Low, 2003)

**Delta C** - Standard deviation on consonantal (intervocalic) segments ignoring word boundaries (i.e., consonant at the end of one word was summed with onset consonant at the beginning of the next word ignoring silence separating the two words if present; see Grabe & Low, 2003).

**Delta V** - Standard deviation in vocalic interval segments also ignoring word boundaries.

offset of last syllable subtracted from the onset of the first syllable - Number of syllables divided by the total duration (offset of last syllable subtracted from the onset of the first syllable; see Grabe & Low, 2003).

*Music Information Retrieval*

**Spectral Flux: Overall** - the average distance between successive frames in a spectrum. This metric results in larger coefficients when there are important moments in time with high contrast in the spectrum.

**Spectral Flux: Sub-band**: Sub-band spectral flux was calculated in the same manner as spectral flux except flux is calculated separately for each of 10 equally-spaced frequency bands along the spectrum. Only the first 9 bands were used as some stimuli had little to no energy in the 10th band of the spectrum.

**Pulse Clarity: Maximum** - metric of rhythmic clarity derived from autocorrelation *mirtempo* function, returning the *maximum* correlation value in the mirtempo autocorrelation curve (Lartillot et al., 2008)

**Pulse Clarity: Minimum**- metric of rhythmic clarity derived from autocorrelation *mirtempo* function, returning the *minimum* correlation value in the mirtempo autocorrelation curve (Lartillot et al., 2008)

**Tempo: Autocorrelation -** estimation of the speed of the onsets by detecting periodicities from the autocorrelation of the onset detection curve calculated via *mironsets* using the envelope,

**Tempo: Spectrum** - spectral decomposition of the onset detection curve calculated via the envelope (mironsets).

**Tempo: Spectral Flux -** estimation of the speed of the onsets by detecting periodicities from the autocorrelation of the onset detection curve calculated via *mironsets* using spectral flux,

*Music-inspired*

**Integer Multiples** - Following the pairwise ratio calculations outline in Roeske and colleagues (2020), we estimated the small integer ratio bias by finding the durations between 3 consecutive syllable onsets (two intervals) at a time for the full sentence. The “cycle duration” is the sum of the two intervals, and the ratio is calculated by taking the first interval in the pair and dividing it by the cycle duration. The proportion of intervals related to each other by small integer ratios (.25, .33, .5, .66, .75, where .5 is isochronous or 1:1 relationship between the 2 intervals) was estimated with a .05 bin around the small integer (.025 on either side of the small integer ratio). All ratios falling within these ranges were coded as small integer ratios and outside of those bands, large integer ratios. The proportion of cycles in small integer ratios was used as the per sentence metric.

**Asynchrony** - For all utterances, base unit syllables were identified by choosing the shortest syllable length with at least one other syllable of a similar size. The final base unit for each utterance was calculated using the average of all the identified base units. The base unit was used to create a metronome aligned to the first base unit in the sentence, but creating a metronome for the full length of the stimulus. Note that if syllable durations were related to each other in a metrical or integer multiple framework the shortest syllable should provide a clock-like (Povel & Essens, ) metronome that should align well with all onsets in the utterance. Asynchrony was estimated by calculating the average absolute value of the difference between each syllable onset and the estimated onsets from the shortest-syllable-derived metronome.

**Asynchrony Variability** - the standard deviation of the absolute value of the difference between each syllable onset and the estimated onsets from the shortest-syllable-derived metronome.

**Signed Asynchrony -** the average signed difference between each syllable onset and the estimated onsets from the shortest-syllable-derived metronome, with negative values coming earlier than the metronome and positive values coming after the onset.

**Signed Variability -** the standard deviation of the signed difference between each syllable onset and the estimated onsets from the shortest-syllable-derived metronome, with negative values coming earlier than the metronome and positive values coming after the onset.
